# Back to the future of psychoneuroimmunology: Studying inflammation-induced sickness behavior

**DOI:** 10.1016/j.bbih.2021.100379

**Published:** 2021-10-24

**Authors:** Julie Lasselin

**Affiliations:** a Stress Research Institute, Department of Psychology, Stockholm University, SE-106 91, Stockholm, Sweden; b Division of Psychology, Department of Clinical Neuroscience, Karolinska Institutet, Stockholm, Sweden; c Osher Center for Integrative Medicine, ME Neuroradiologi, Karolinska Universitetssjukhuset, Stockholm, Sweden

**Keywords:** Sickness behavior, Experimental endotoxemia, Lipopolysaccharide, Motivation, Inflammation-associated depression, Inter-individual differences

## Abstract

What do we know about sickness behavior? In this article, I guide you through some of the complexity of sickness behavior occurring after an immune challenge. I highlight the many features of behavioral and affective changes induced during experimental endotoxemia in humans, and describe how little we know about many of these features. I argue that we need to dismantle the components of inflammation-induced sickness behavior, and study each component in detail. I also point out the large inter-individual differences in inflammation-induced behavioral and affective changes, and the fact that psychosocial factors likely interact with inflammation to shape inflammation-induced sickness behavior. PNI clearly lacks investigations of the vulnerability and resilient factors underlying the inter-individual variability in sickness behavior. Throughout the article, I base my argument on my published articles, and provide concrete examples from my experience and the data that I have collected over the past 10 years. Given the relevance of inflammation-induced sickness behavior for inflammation-associated depression and for how people react to infections, I encourage current and future psychoneuroimmunologists to return towards basic science of sickness behavior.

## Highlights

• Inflammation-related sickness behavior is relevant for inflammation-associated depression
• The many features of sickness behavior should be investigated in detail
• There are large inter-individual variability in sickness behavior
• Vulnerability and resilient factors predicting sickness responses are little known
• I call for a return towards basic science of sickness behavior.

Sick individuals experience weakness, malaise, listlessness, and inability to concentrate. They become depressed and lethargic, show little interest in their surroundings, and stop eating and drinking. Their range of preoccupations is limited to their own body and the suffering they are experiencing. This constellation of nonspecific symptoms is collectively referred to as ‘sickness behavior.’

Robert Dantzer, 2001

Robert Dantzer is one of the fathers of Psychoneuroimmunology (PNI) and a truly inspiring pioneer for many of us in this field. His definition of sickness behavior follows the description of the adaptive behavioral changes occurring in sick animals by Benjamin Hart (1988). This definition appears clear, and you could accept it without batting an eye. However, picture yourself and people from your entourage sick, and reconsider this definition again. Does everyone you know show all these signs of sickness, and at every episode of infection? Would you show all these signs if you knew you would be well taken care of? Can you think about any other signs of sickness that are not depicted here?

**Fig. 1:**
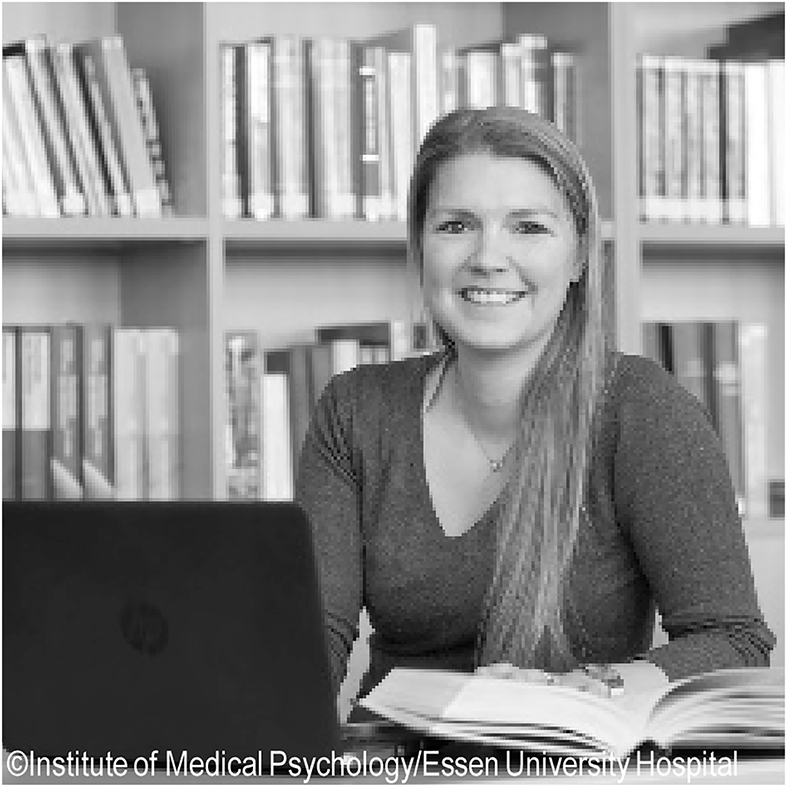
**Julie Lasselin.** Julie Lasselin obtained her PhD from the University of Bordeaux in 2012 with Dr. Lucile Capuron as her supervisor. She was then a post-doctoral fellow with Prof. Mats Lekander and Prof. John Axelsson in Stockholm (Sweden) in 2014–2015; and with Prof. Manfred Schedlowski in Essen (Germany) in 2016–2017, funded by the Alexander von Humboldt foundation. Dr. Lasselin is currently a researcher at the Stress Research Institute, Stockholm University, and at the Department of Clinical Neuroscience, Karolinska Institutet (Stockholm, Sweden). Her work includes basic science research using clinical and experimental models in humans, and aims to characterize in details the overt and subjective behavioral changes induced by inflammation in humans, investigate the adaptive relevance of sickness behavior, analyze the psychological and biological factors that interact with cytokines to affect the brain and behavior, and the underlying mechanisms. Dr. Lasselin also investigates how overt changes in behavior during inflammatory sickness affect the relationship with others and the care one receives, and how this in turn modulates health outcomes. Dr. Lasselin is a member of the editorial board of *Brain, Behavior and Immunity* and *Brain, Behavior, and Immunity – Health,* and is part of the steering committee and webmaster of the European Psychoneuroimmunology Network (EPN, https://pnieurope.eu).

The description of sickness behavior has certainly moved the field of PNI forward, in particular with respect to Immunopsychiatry (Dantzer et al., 2008). However, although Dantzer left the door open for more complex interpretations of sickness behavior by also defining sickness behavior as “the expression of a central motivational state that reorganizes the organism's priorities to cope with infectious pathogens” (Dantzer, 2001), it seems that the more simple definition above mainly remained in PNI. In this article, I will guide you through some of the complexity of sickness behavior. I will focus on inflammation-induced sickness behavior in humans, mainly because this is my area of expertise (Fig. 1), but behavioral changes in sick animals of various species likely show similar complexity (Lasselin et al., 2020c; Lopes et al., 2021).

## 1 Why studying inflammation-induced sickness behavior?

The study of sickness behavior induced by acute activation of the immune system is central in PNI (Dantzer and Kelley, 2007) and crucial to understand inflammation-associated depression (Lasselin et al., 2020b). Researchers in PNI have established that inflammatory cytokines trigger the behavioral and affective changes observed in sick individuals (Dooley et al., 2018; Schedlowski et al., 2014), and have uncovered some of the brain mechanisms underlying such effects (Kraynak et al., 2018). Given the resemblance between many aspects of sickness behavior and major depression, and the fact that a large proportion of patients receiving long-term treatment with cytokines such as interferon-α become clinically depressed, the relevance of inflammation-induced sickness behavior for depression became obvious for several leaders in the field (e.g., Capuron and Miller, 2004; Dantzer et al., 2008; Savitz and Harrison, 2018). This led to the field of Immunopsychiatry (Pariante, 2019), which focuses on the role of the immune system in the development and maintenance of psychiatric diseases. Researchers in Immunopsychiatry have, for instance, demonstrated the role of inflammation in the development of clinical depression (e.g., Bell et al., 2017; Khandaker et al., 2014), and the existence of a specific subgroup of “inflamed” depressed patients that would likely benefit from anti-inflammatory therapies (Raison et al., 2013).

If we are to understand inflammation-associated depression, we thus need to understand how cytokines affect the brain and behavior (Lasselin et al., 2020b), but not only. We also need to clarify to which extent knowledge about inflammation-induced sickness behavior is relevant for Immunopsychiatry. For instance, do motivational changes observed during acute inflammation match consistently those observed during chronic low-grade inflammation, which is characteristic of inflammation-associated depression? Furthermore, we need to better describe and compare sickness behavior between humans and animals (Lasselin et al., 2020c) to specify how translational is inflammation-induced sickness behavior. Another important point is to understand the shift between acute, adaptive, inflammation-induced sickness behavior to long-term, maladaptive neuropsychiatric symptoms associated with chronic inflammation. Some immunopsychiatrists have started to investigate this issue (see for instance, Capuron and Ravaud, 1999; Dowell et al., 2016), but we would benefit from characterizing the shared behavioral features, and differences, between inflammation-induced sickness behavior and neuropsychiatric symptoms in chronic inflammatory conditions. Finally, we need to determine the factors that render individuals more vulnerable, or more resilient, to inflammation-induced behavioral and affective changes.

Studying sickness behavior is also relevant to understand how people react to infectious agents, and why they react so differently. For instance, some people cannot do anything but rest when suffering from a common cold, while others can go on with their life even with a stronger respiratory infection. This issue has been featured by the current COVID-19 pandemic: during this pandemic, individuals who felt sick were asked to stay home to limit the spread of SARS-CoV-2; but then, how to handle infected and possibly contagious, but asymptomatic, individuals? It is thus important to understand what make people more or less vulnerable to *feeling* sick when inflamed.

## 2 Back to the roots of PNI: towards a better characterization of inflammation-induced sickness behavior

The model of experimental endotoxemia is central when studying inflammation-induced sickness behavior (Lasselin et al., 2020b). I have personally a predilection for this model, which consists in injecting intravenously a bacterial endotoxin (lipopolysaccharide, LPS) to healthy volunteers, triggering a systemic inflammatory response and sickness behavior for 4–5 ​h. Participants recover quite quickly and are discharged 5–7 ​h post-injection. Thus, this model allows studying the development of sickness behavior as well as its recovery, in a short amount of time. This model was used in PNI research for the first time in 1993 by Thomas Pollmächer to investigate the effect of inflammation on sleep (Pollmacher et al., 1993), then joined by Raz Yirmiya to investigate inflammation-induced emotional and cognitive changes (e.g., Reichenberg et al., 2001). A number of groups have since then used this model in PNI research (Dooley et al., 2018; Lasselin et al., 2018a; Schedlowski et al., 2014).

The first article that came out of my first study using the model of experimental endotoxemia made me reconsider the concept of inflammation-induced sickness behavior: I realized how little we know about sickness behavior in humans. We used an effort-based decision-making paradigm, the EEfRT (Effort Expenditure for Rewards Task, Treadway et al., 2009), to investigate the effort-reward balance during acute inflammation, such as conducted previously in rodents (e.g., Merali et al., 2003; Nunes et al., 2014). In accordance with rodent studies, we hypothesized that individuals would show a reduced willingness to make an effort to obtain a (monetary) reward during LPS-induced sickness. We were quite surprised when the data indicated that sick individuals exhibited a *stronger* willingness to make a high effort to obtain a higher monetary reward, instead of choosing to do a low effort and obtain a lower reward. This effect appeared, however, only when the probability to obtain the reward was very high (88%) (Lasselin et al., 2017). Interestingly, another study using the model of experimental endotoxemia with another effort-based decision-making paradigm was published a few months later (Draper et al., 2018). In this study, Marieke van der Schaaf's research group reported no change in reward sensitivity (i.e., sick participants liked money similarly as when healthy), as in our study, but reported a *reduced* willingness to make an effort to obtain a monetary reward in sick individuals compared to individuals who had received a placebo. There is no reason to believe that one of the studies was not well conducted, or that data were not well analyzed. Instead, I believe that these two studies, conducted with the same dose of LPS (although using different procedures, and probably different endotoxin lot), feature the complexity of sickness behavior. When sick, you will show a reduced motivation for monetary reward if the other choice from making an effort would be to rest. However, if you are forced to make an effort, as it was the case in our study (high-effort/high-reward or low-effort/low-reward), you might choose to redirect your effort towards something comforting, in particular if your chances to get this reward is high (88% probability). A study in rodents conducted by Elisabeth Vichaya, Sarah Hunt, and Robert Dantzer, a few years before (Vichaya et al., 2014), supports this notion. In this study, although sick rodents showed an overall reduced willingness to expend effort to obtain food reward (pellets and chocolate), when they did make an effort, they redirected it towards the preferred reward (i.e. chocolate).

The intensity of the inflammatory and sickness response might also influence the redirection of motivational goals. In a more recent study, Julienne Bower's research group used the EEfRT in individuals who had been vaccinated against influenza the day before (Boyle et al., 2019). Influenza vaccine induces a very mild inflammatory response, and no noticeable sickness symptoms. The authors could nevertheless establish that a higher inflammatory response after vaccination was related to a lower willingness to make a high effort in order to get a higher reward. In other words, individuals with higher inflammatory response to influenza vaccine preferred to make low effort even if it meant having lower monetary reward. A milder inflammatory stimulus, but also small changes in the paradigm (use of dominant hand for the high effort task instead of the non-dominant hand, shorter task duration), could explain the difference between these findings and ours. Another important factor that could affect motivational outcomes is the type of reward. All the studies described above used monetary rewards, which, although quite comforting, do not provide a direct benefit to sick individuals. If you consider that, rather than obtaining a monetary reward, sick individuals are asked to make an effort in order to being able to sleep or rest (Miller, 1964), or to obtain care, the effects might differ substantially. With respect to the latter, Naomi Eisenberger and her colleagues have described that sick individuals show an increased brain sensitivity to social rewards, such as viewing close others and hearing positive social feedback (Eisenberger et al., 2016). They argue that this would provide the possibility to the sick individual to approach potential caregivers and obtain support from others. Altogether, sickness-related motivational goals during sickness will compete with other motivational goals; and the context (Lopes, 2014), the paradigm parameters, and most probably the type of reward will affect the willingness of sick individuals to expend effort. Therefore, we need to explore motivational changes during sickness in more details.

Change in motivated behaviors is not the only feature of sick individuals, who also *feel* sick, and in particular tired, fatigued, and sleepy. Fatigue is a symptom that appears highly sensitive to inflammation (and is highly common in clinical populations), but still remains poorly investigated in relation to sickness behavior. Fatigue is however central in sickness, since it will lead sick individuals to rest, allowing preserving body energy. In a recent paper, we describe the kinetic of fatigue and sleepiness in four studies that used experimental endotoxemia (Lasselin et al., 2020a). We show that the development of fatigue and sleepiness closely parallels the inflammatory response, and both are strongly related. However, fatigue was measured very broadly, as in most studies assessing fatigue during experimental endotoxemia (e.g., DellaGioia et al., 2013; Hannestad et al., 2011), for instance by asking participants to describe how they felt, from “absence of fatigue” to “severe fatigue”, or by using the fatigue dimension of the Profile of Mood States. However, fatigue is a multidimensional symptom, defined as “the failure to initiate and/or sustain attentional tasks and physical activities requiring self-motivation” (Chaudhuri and Behan, 2000). Hence, it includes a *mental* dimension, a *physical* dimension, and a *motivation* dimension. As my colleagues and I have argued (Karshikoff et al., 2017), given that the dimensions of fatigue likely relate to different neuronal and even eventually metabolic mechanisms (Dantzer et al., 2014; Lacourt et al., 2018), we cannot understand the role of inflammation in fatigue without assessing these dimensions. Another question is how the subjective feeling of fatigue relates to objective measurements. As mentioned above, although individuals in our study were feeling sick and tired, they could overcome this feeling and get more motivated to obtain a high monetary reward (Lasselin et al., 2017). Furthermore, in our recent study, individuals did not show psychomotor slowing in a simple reaction time task and in a go/no-go task after receiving LPS compared to placebo, although they perceived performing less well (Handke et al., 2020). Given that only objective measurements can be used in animals, it seems important to understand how closely (and in which context) subjective feelings relate to objective measurements (e.g., locomotor activity, cognitive performance, motivated behavior).

The subjective feeling that has been the most extensively studied during experimental endotoxemia is probably negative mood. Many psychoneuroimmunologists have assessed the brain underpinnings of negative mood during sickness, with the aim to understand the mechanisms underlying inflammation-associated depression. They have suggested that inflammation induces a negative bias, with an increased sensitivity to negative stimuli (Benson et al., 2017a; Eisenberger et al., 2009; Harrison et al., 2016), explaining the development of negative mood. Experimental endotoxemia also triggers state anxiety quite strikingly (Lasselin et al., 2016), although this symptom has been overall less investigated. However, challenging the notion of a clear association between inflammation and negative emotions, the group of Robert Dantzer could not find evidence of a negative bias in mice in a recent study (Casaril et al., 2021). Furthermore, we also tested this “negative bias” assumption in a recent pilot study, by assessing emotional regulation (cognitive reappraisal of emotions) during experimental endotoxemia (Hansson et al., 2021). Contrary to what we expected, participants in the LPS condition reported *greater* success in down-regulating their emotions towards negative stimuli compared to the placebo condition. The two latter studies thus call for a more thorough investigation of the emotional changes during sickness.

Beyond motivated behaviors and subjective feelings, I would argue that we should consider other changes that manifest during sickness and that could affect the sickness response and/or the interaction of the sick individual with others, as part of sickness behavior. For instance, we have shown that participants during experimental endotoxemia had a more rigid way of walking, with shorter, slower, and wider strides, less arm extension and knee flexion, and a head tilting downwards (Lasselin et al., 2020d). These changes in gait parameters could relate to pain and malaise, and speculatively also partly to energy preservation: if you walk more slowly and with more restricted movements, it will consume less energy while still allowing you to reach your goal. In addition, peers could also use these gait changes to recognize that you are sick, and, thus, avoid you to prevent contagion, or approach you to provide care. Gait changes can thus arguably be included in “sickness behavior”. We have also shown that other manifestation of sickness, such as changes in facial cues (e.g., paler lips, droopier corners of the mouth (Axelsson et al., 2018b), and in body odor (Regenbogen et al., 2017)), can be used by others to recognize a sick person. Experimental endotoxemia also induces a strong increase in yawning frequency (Marraffa et al., 2017). Although the function of yawning remains unclear, others could interpret it as a sign of malaise and potentially sickness. The recognition of sick individuals by the persons in their surroundings will affect social interactions. Hence, the sick individual's peers can then choose to provide care and comfort, but also to avoid or isolate the sick individual in order to prevent infection to other members of the group (Dantzer, 2021). Changes in social behaviors of the sick individual's peers can also, ultimately, affect behaviors and feelings of the sick individual.

Another aspect of sickness behavior is how sick individuals *express* how they feel. In one of my favorite studies, we have assessed how sick men and women expressed their malaise through verbal complaints, moans, and sighs/deep breaths (Lasselin et al., 2018b). We observed very low frequency of verbal complaints and moans, but a strong increase in the frequency of sighs/deep breaths after the injection of LPS, however in men only. In fact, 85% of the men compared to 33% of the women exhibited more than 36 sighs/deep breaths during the 3 ​h of the peak sickness response. Importantly, men and women reported similar intensity of subjective sickness feelings, indicating that the expression of sickness malaise does not necessarily relate to how people feel. We have suggested that the difference in how men and women expressed their malaise in our study could relate to the notion of “man flu” (Axelsson et al., 2018a).

**Fig. 2:**
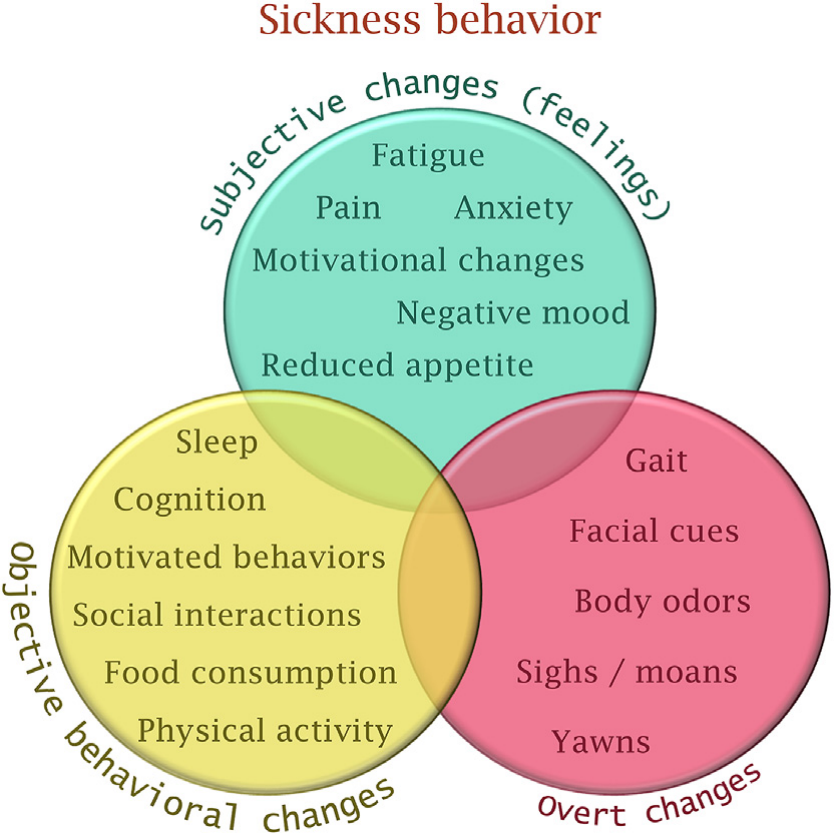
**The many features of inflammation-induced sickness behavior.** Inflammation-induced sickness behavior is highly complex, and encompasses subjective feelings, objective behavioral changes, and, I argue, overt manifestations of sickness. All the features will not be observed in all sick individuals, and many variables can modulate the expression of sickness behavior. Furthermore, the different aspects of sickness behavior do not necessarily correlate. For instance, a sick individual can *feel* extremely bad, yet not exhibit objective changes or overt manifestations of sickness.

To conclude, sickness behavior induced by acute inflammation contains many features, and we need to dismantle the components of sickness behavior, and investigate each component in detail, such as what has been proposed for inflammation-associated depression (Capuron and Miller, 2004; Dantzer et al., 2008; Lasselin, 2020; Moriarity and Alloy, 2020). In Fig. 2, I describe some of the features that I believe should be included in the concept of “inflammation-induced sickness behavior”. I am well aware that not everyone in PNI would accept all these features. I would answer that it is time to start a discussion to determine precisely what sickness behavior is – and eventually extend the concept of sickness behavior to a larger concept that includes all the features of the sick individual, including subjective feelings but also objective behavioral changes and overt changes – in PNI, and beyond (Konsman, 2021).

## 3 To be sick or not to be sick: inter-individual variability in inflammation-induced sickness behavior

### 3.1 Sickness behavior varies highly between sick individuals

**Fig. 3:**
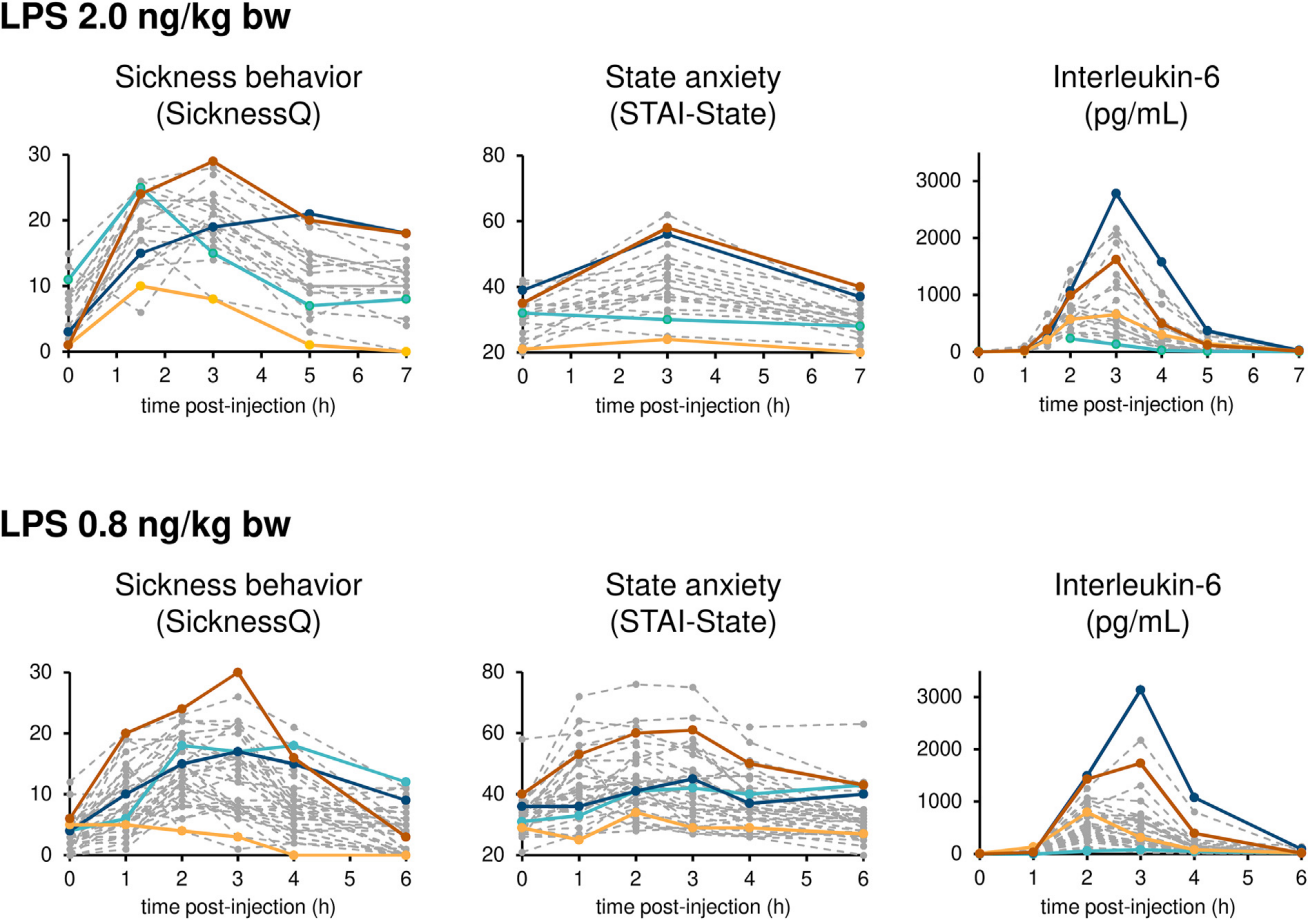
**Individual responses during experimental endotoxemia.** The figure illustrates individual responses to an injection with lipopolysaccharide (LPS) in two studies, one using a dose of 2.0 ​ng/kg body weight (upper panels) and one using a dose of 0.8 ​ng/kg body weight (lower panels). In both studies, we can notice a large inter-individual variation, both regarding the inflammatory response, as illustrated with interleukin-6 concentrations, and the subjective sickness response, as illustrated with the Sickness Questionnaire (SicknessQ (Andreasson et al., 2016)), and the State part of the State-Trait Anxiety Inventory (STAI-State, (Spielberger et al., 1979)). Furthermore, the data illustrate the potential involvement of other factors than inflammatory cytokines to shape sickness behavior: the individuals with the weakest SicknessQ response (light orange ) or with the strongest SicknessQ response (dark orange ) do not have the lowest or highest immune response; and the individuals with the weakest interleukin-6 response (light blue ) or with the strongest interleukin-6 response (dark blue ) do not exhibit a particularly low or strong sickness response. (For interpretation of the references to colour in this figure legend, the reader is referred to the Web version of this article.)

We all react differently when sick: some of us feel terrible at every little infection, while others, although sometimes clearly infected (e.g., coughing), do not *feel* this infection. I had the opportunity to witness this in the studies I have conducted using experimental endotoxemia, the first study being particularly striking with this respect. We had used a relatively high dose of LPS (2.0 ​ng/kg body weight), which induces strong flu-like symptoms. The large majority of the participants felt extremely sick after the LPS injection, and reported feeling much worse than usual when sick. Still, a few of them did not feel that much sick and reported feeling as sick as usual, or even better than usually when sick, and I could actually myself not *see* what they had been injected with (I was blind to the condition). I thought that we had made a mistake and/or that their immune system did not react to the bacterial endotoxin. However, cytokine concentrations did increase for these participants, and even stronger than for others. Fig. 3 illustrates individual inflammatory and subjective sickness responses from this study and another one with a lower dose of LPS (0.8 ​ng/kg body weight, Lasselin et al., 2020e). When observing these data, it becomes obvious that the inflammatory response does not explain all the variability in subjective sickness feelings. Although inflammatory cytokines certainly trigger behavioral and affective changes, other factors probably interact with inflammation to shape sickness behavior, in its intensity but also its recovery.

### 3.2 Potential psychosocial factors shaping inflammation-induced sickness behavior

When discussing about the role of psychosocial factors shaping sickness behavior, I often face answers such as “other *physiological* factors than cytokine concentrations could explain the variability, such as functions of cytokine receptors or of brain immune cells”. I certainly agree, many physiological factors can affect sickness behavior (e.g., activity of transcription factors (Cho et al., 2019), white matter volume (Månsson et al., 2021)), and these factors should be investigated. I argue, however, that psychosocial factors can probably also influence sickness behavior.

Researchers in the placebo field are well aware of the importance of psychosocial factors for health outcomes. Two main mechanisms are acknowledged to be involved in the placebo effect, namely classical conditioning and individuals’ expectations (Finniss et al., 2010). In the context of inflammation-induced sickness behavior, I am highly interested in the latter, i.e. how do expectations based on previous experience, knowledge, and information, affect how we feel when sick. In the first study assessing this question (Lasselin et al., 2018c), we have asked participants how sick they were expecting to be, before they received the LPS injection. The large majority were expecting to feel as usually when sick, and, thus, did not expect to feel as sick as the dose of 2.0 ​ng/kg of LPS would induce. Interestingly, we observed that participants who expected to *not* feel much sick reported *more* anxiety, negative affect, and fatigue, after the injection of LPS. In other words, those who expected to feel *very* sick did not feel so anxious, sad, and fatigued during experimental endotoxemia. Our data also suggest that this effect might relate to *prediction error*s, with stronger sickness outcomes being predicted by a larger discrepancy between what was expected and the intensity of the immune signal. Such effect has actually been proposed previously in the placebo literature, for instance with respect to pain (Buchel et al., 2014). Our findings strongly indicate that sickness behavior is not only determined by the intensity of the immune response, but is modulated by top-down processes. In particular, I believe that fatigue and mood responses during sickness are reactions to the integration of bodily signals in the brain (as proposed for emotions by Lisa Feldman Barrett, 2017), and as such are highly sensitive to top-down processes such as predictions.

Only a few groups in PNI have investigated other potential psychosocial factors contributing to inter-individual variability of sickness behavior. Among the factors that are likely to modulate sickness behavior, we can cite: gender (Lasselin et al., 2018a); baseline psychological state such as state anxiety (Lasselin et al., 2016), negative affectivity (Benson et al., 2017b; Harper et al., 2017; Lacourt et al., 2015), neuroticism (Cvejic et al., 2019), perceived stress (Irwin et al., 2019), and sleep disturbances (Cho et al., 2016); and socioeconomic disadvantage (Cvejic et al., 2019). Sociocultural factors such as stoic endurance of pain and familism might also be factors of interest, as they were related to the intensity of sickness behavior in a national U.S. sample (Shattuck et al., 2020).

Altogether, many psychosocial factors could interact with inflammation to modulate sickness behavior, but these remain largely under investigated. One question that remains unanswered regards the universality of the inter-individual variability in sickness behavior, i.e. would someone showing strong sickness behavior after a bacterial stimulus (e.g. LPS) also be very sensitive to other types of pathogens (viruses, parasites, fungi). Importantly, if we define the factors underlying the vulnerability and resilience to inflammation-induced behavioral and affective changes, we can develop therapies targeting them (e.g., cognitive behavioral therapies), and test such therapies using the model of experimental endotoxemia (Lasselin et al., 2020b).

## 4 Call for the future of PNI

If I ask you now, what do we really know about inflammation-induced sickness behavior? I guess – I hope – I will have convinced you that we do not know enough. We obviously know some, but there are many aspects of sickness behavior that remain unexplored (see **2.** and Fig. 2) and we know only little about the factors shaping inter-individual variations in sickness behavior (see **3.** and Fig. 3). Researchers in PNI have overall neglected the basic science of sickness behavior, maybe because of the misconceived notion that it is “not mechanistic enough” (Konsman and Reyes, 2020). A detailed understanding of inflammation-induced sickness behavior is nevertheless necessary (see **1.**), and I urge present and future psychoneuroimmunologists to help me in this endeavor.

## Funding

Research work described in this article and Julie Lasselin are supported by the 10.13039/501100004359Swedish Research Council [grant numbers 2017-02629 to Mats Lekander; 421-2012-1125 and 2016-02742 to Mats J Olsson; 2020-01606 to Julie Lasselin], the Swedish Foundation for Humanities and Social Sciences [grant number P12-1017 to Mats J Olsson], 10.13039/501100013346Stockholm Stress Center, and the 10.13039/100005156Alexander von Humboldt foundation [grand number 1156790 to Julie Lasselin]. The funders were not involved in the preparation of this article.

## Declaration of competing interest

Declare no conflict of interest.
